# Topological Weyl and Nodal Line Half-Metals in Two-Dimensional van der Waals Material EuOX (X = F, Cl, Br, I)

**DOI:** 10.3390/ma19102154

**Published:** 2026-05-21

**Authors:** Sheng-Hsiung Hung, Horng-Tay Jeng

**Affiliations:** 1 Department of Physics, National Tsing Hua University, Hsinchu 30013, Taiwan; 2 Physics Division, National Center for Theoretical Sciences, Taipei 10617, Taiwan; 3 Institute of Physics, Academia Sinica, Taipei 11529, Taiwan; 4 Research Center for Semiconductor Materials and Advanced Optics, Chung Yuan Christian University, Taoyuan 32031, Taiwan

**Keywords:** 2D magnet, half-metal, 2D topological materials, Weyl point, nodal line, first-principles calculation, DFT+U, Curie temperature, magnetic anisotropy

## Abstract

Two-dimensional magnetic materials with an atomic-thick layered structure have long been a focal point in condensed matter physics owing to the intrinsic long-range magnetic order, the monolayer limit with high tunability and potential in nano-scale spintronics. In this study, we report a novel family of two-dimensional (2D) materials, EuOX (where X = F, Cl, Br, I), displaying 2D half-metallicity with topological properties. Specifically, the spin-up conducting channel demonstrates metallic behavior, while the spin-down channel exhibits an insulating band gap at the Fermi level. The presence of Weyl points and nodal lines coexists in the spin-up conductive channel of EuOX monolayers. These topological properties, alongside the half-metallic behavior, evolve systematically with the strong-correlation Hubbard U. These findings provide crucial insights into the design of 2D topological spintronic devices, offering a promising platform for future spintronic applications in the nano scale.

## 1. Introduction

Since the successful isolation of graphene in 2004 [1], research on two-dimensional (2D) materials has formally commenced. However, most early systems lack intrinsic magnetism [2]. According to the Mermin–Wagner theorem, long-range magnetic order in two-dimensional systems is highly susceptible to thermal fluctuations. Nevertheless, magnetic anisotropy (MA) can effectively suppress these fluctuations, enabling the stabilization of magnetism in 2D materials at finite temperatures [2,3]. Despite this progress, the majority of early systems remain short of intrinsic magnetic properties [2]. Since 2016, there have been numerous reports of 2D magnetic materials that can be exfoliated similar to graphene. Subsequently, active control and device applications emerge; for example, bilayer CrI_3_ can achieve electric switching between antiferromagnetic and ferromagnetic interlayer states via gate voltage modulation [4,5], while the Curie temperature (T_C_) of Fe_3_GeTe_2_ can be tuned close to room temperature through ion-gate regulation [6]. Furthermore, monolayers of FeX_2_ and FeCl_2_, possessing intrinsic half-metallicity, have been proposed as ideal electrode materials for spintronic devices [7,8]. Scientists can further induce exchange coupling in non-magnetic layers and achieve dynamic control over spin and valley degrees of freedom through the spin–orbit torque mechanism [9,10,11]. To address the challenge of low T_C_, theoretical studies predict high-temperature ferromagnetic monolayers for MnNX and CrCX families with T_C_ values approaching 500 K [12,13,14]. Additionally, the Gd_2_B_2_ structure demonstrates exceptional thermal stability [15]. In terms of application prospects, 2D magnetic systems, characterized by protected chiral edge states via bulk–edge correspondence, are considered pivotal materials for developing low-energy-consumption electronic interconnects, quantum metrology standards, and fault-tolerant topological quantum computing [16,17]. However, realizing device applications still faces significant challenges, primarily including low operational temperature scales, internal material disorders and defects, environmental sensitivity, and narrow process windows [6,18,19].

Theoretical investigations have unveiled exciting possibilities, with monolayers of rare-earth dihalides (GdX_2_, where X = I, Br, Cl, F) and Janus structures like GdClF demonstrating the potential for high-temperature ferromagnetism. Notably, theoretical predictions suggest Curie temperatures approaching 443 K for some materials, while the Gd_2_B_2_ structure exhibits exceptional thermal stability with predicted Curie temperatures exceeding 550 K [15,20,21]. Furthermore, the rich phase diagram of rare-earth compounds has been theoretically predicted. Families such as RI_2_ (where R represents a lanthanide) and RICl_3_ (e.g., GdI_3_, GdCl_3_) display a spectrum of magnetic ground states, ranging from Néel-type antiferromagnetism to ferromagnetic semiconductors. These systems offer fertile ground for studying phenomena like magnetic phase transitions, multiferroicity, and spin–valley coupling, potentially enabling the manipulation of ferrovalley order through strategic intercalation (e.g., with Li or Mg) or controlled stacking configurations [20,21,22].

Experimental progress is equally impressive. The RTe_3_ (where R is a lanthanide) family has emerged as a premier platform for studying electron correlation and topological phenomena in van der Waals materials. For instance, GdTe_3_ demonstrates ultrahigh electron mobility (>60,000 cm^2^/V·s) and can be exfoliated down to few atomic layers [23]. In contrast, systems like NdTe_3_ and DyTe_3_ reveal thickness-dependent magnetic properties and host complex orders such as incommensurate helimagnetism coupled with charge density waves (CDWs) [24,25]. Beyond intrinsic properties, interface engineering has proven powerful. The proximity effect enables the transfer of magnetic order and enhanced spin–orbit torque functionality across material boundaries. Heterostructures like graphene/EuS and EuS/TMD have successfully demonstrated strong exchange bias and significant valley polarization, paving the way for advanced spintronic applications [9,26].

The research on two-dimensional magnetic topological materials integrates magnetic order with non-trivial band topology, providing an ideal platform for exploring exotic physical phenomena such as the Quantum Anomalous Hall Effect (QAHE) [17,19]. Since the first experimental observation of the QAHE in 2013 [17], the research field has evolved into three main directions: First, magnetically doped topological insulators exhibit high-fidelity QAHE but are limited by ultra-low temperatures (milli-Kelvin range) due to dopant disorder [17]. Second, intrinsic magnetic topological insulators (MTIs), exemplified by MnBi_2_Te_4_, utilize the switching of magnetic order in van der Waals heterostructures with odd/even layer numbers to realize QAHE, axion insulator (AI) phases, and related magnetoelectric effects [17,27]. EuSn_2_As_2_ has been identified as an intrinsic magnetic topological insulator, while EuCd_2_As_2_ exhibits properties consistent with antiferromagnetic Dirac semimetals [28,29,30]. Moreover, kagome lattice materials like YbMnBi_2_ and TbMn_6_Sn_6_ host exotic quantum phenomena, including manifestations of topology under high magnetic fields and evidence of Weyl fermion excitations [31,32,33]. Third, twistronics introduces moiré superlattice engineering, enabling dynamic control of Berry curvature through twist angles to induce Chern insulating states and even fractional quantum Hall states (FQH) at zero magnetic field [34,35]. Furthermore, topological semimetals like the kagome magnet Fe_3_Sn_2_ and Weyl semimetal Co_3_Sn_2_S_2_ demonstrate magnetically tunable Weyl fermions and large anomalous Hall conductance [36,37]. Single-layer MnN has been predicted to host spin-polarized nodal lines as a two-dimensional semimetal [12]. Concurrently, twistronics introduces a new dimension by revealing ferrimagnetic and antiferromagnetic coexistence in slightly twisted CrI_3_ [35,38].

On the other hand, the exploration of lanthanide rare-earth materials with strong localized 4f electron configurations has emerged as a significant frontier in two-dimensional magnetism research. Compared to conventional 3d transition metals, the unique electronic structure of lanthanides, characterized by the shielding effect of the outer 5s/5p orbitals on the inner 4f electrons, enables remarkable phenomena including exceptionally high localized magnetic moments, long dephasing times, and pronounced spin–orbit coupling (SOC). These properties are crucial for stabilizing long-range magnetic order in low-dimensional systems and overcoming the limitations posed by insufficient magnetic anisotropy [20,39,40]. Researchers have developed a lanthanum europium oxychloride (La_x_Eu_1−x_OCl) solid-solution catalyst, discovering that LaOCl can act as a chlorine buffer and work synergistically with active Eu sites to accelerate the kinetically hindered chlorination step in methane oxychlorination. This synergistic effect enhances the selectivity of CH_3_Cl production while reducing production costs [41]. Secondly, through salt-assisted chemical vapor deposition (CVD), two-dimensional EuOCl nanosheets have been successfully synthesized. These nanosheets exhibit an extremely narrow photoluminescence spectrum (full width at half maximum of 1.2 meV) at room temperature. The anomalous temperature-dependent optical properties have been attributed to the Judd–Forster effect, opening new avenues for display and illumination technologies [42]. Finally, first-principles calculations predict that monolayer EuOBr represents a novel two-dimensional ferromagnetic semiconductor. This material demonstrates 100% spin polarization and hosts multiple topological nodal lines and Weyl points protected by mirror symmetry. The topological characteristics exhibit exceptional robustness against spin–orbit coupling, providing a crucial material platform for future spintronic devices and topological research [43].

Collectively, these advancements significantly expand the landscape of two-dimensional magnetic materials. They provide crucial platforms for investigating high-temperature magnetism, manipulating spin and valley degrees of freedom, and exploring fundamental topological phenomena. This rich tapestry of material behaviors offers fertile ground for developing next-generation spintronic devices, ultra-efficient quantum computing components, and novel electronics operating at room temperature [20,39,44]. Despite the significant attention garnered by the topological nodal-line half metal EuOBr [43], there exists a notable demand in the literature regarding systematic studies of its entire series EuOX (where X = F, Cl, Br, I). Although existing studies have reported band structure calculations of the EuOX series using a single fixed Hubbard U value for comparison with optical experiments [45], a comprehensive analysis of their magnetic and topological properties remains absent. Furthermore, the influence of the Hubbard U parameter, a critical descriptor for 4f electron correlation effects, on the electronic structure, structural stability, and magnetic properties of these materials remains inadequately explored. This work aims to address these issues. We systematically investigated the two-dimensional EuOX materials for X = F, Cl, Br, I using first-principles computational methods. Our primary research objectives are:

1. Structural Stability and Exfoliability: To assess the dynamical stability and potential for exfoliation into two-dimensional sheets through phonon band structure calculations.
2. Magnetic Property: To investigate the antiferromagnetic ground state and estimate the magnetic transition Curie temperature using computational methods such as the harmonic approximation and Monte Carlo simulations.
3. Electronic Structure and Hubbard U Dependence: To demonstrate the topological band structures including Weyl and nodal line, and also systematically examine the dependence on Hubbard U and spin orientation.

This systematic computational study seeks to provide a deeper and more comprehensive understanding of the unique behavior of 4f electrons in two-dimensional materials, specifically their electronic structure, structural stability, and magnetic property dependence on Hubbard U. These findings are anticipated to be crucial for the future design and development of functional two-dimensional materials based on rare-earth elements, with significant implications for spintronics and topological research.

## 2. Computational Methods

The electronic structure calculations are performed using the projector augmented wave method with the Perdew–Burke–Ernerhof (PBE) generalized gradient approximation (GGA) as implemented in the Vienna ab initio simulation package (VASP) code (version 5.4.4) [46,47,48,49,50,51]. The pseudopotentials used are Eu (valence configuration: 5s^2^5p^6^4f^7^6s^2^, treating 4f electrons as valence states), O(2s^2^2p^4^), F(2s^2^2p^5^), Cl(3s^2^3p^5^), Br(4s^2^4p^5^), and I(5s^2^5p^5^), taken from the VASP PAW library (version 5.4). The energy cutoff of 700 eV is used for the plane-wave basis expansion with the total energy convergence criteria of 10^−5^ eV per unit cell. We use 12 × 12 × 1 Monkhorst-Pack k-grids over the 2D Brillouin Zone for the self-consistent calculations to assure the convergence. Optimized monolayer structures are obtained with the residual force and stress less than 0.01 eV/A and 1.0 kBar, respectively. The on-site Hubbard U representing strong correlation is incorporated into the electronic structure calculations using the rotationally invariant approach proposed by Dudarev et al. [52], where only the effective parameter $U_{\mathrm{eff}}=U-J$ (the difference between the Coulomb U and exchange J parameters) is considered. The range of U = 0–8 eV is chosen to systematically cover the physically relevant regime for Eu 4f electrons: experimental and theoretical studies of Eu-based compounds report U_eff_ values in the range of 4–8 eV [43,45,53,54,55,56], revealing non-consensus on the U values. Therefore, we perform a systematic analysis with U spanning from 0.0 to 8.0 eV in this work to ensure a comprehensive evaluation and to provide a reliable reference for future experimental comparisons. The phonon band calculations are performed using the QUANTUM ESPRESSO code with the GGA PBE norm conserving pseudopotential [57] without the Hubbard U correction. The pseudopotentials used are Eu (valence configuration: 4f^7^6s^2^), O(2s^2^2p^4^), F(2s^2^2p^5^), Cl(3s^2^3p^5^), Br(4s^2^4p^5^), and I(5s^2^5p^5^). The cutoff energy of 90 Ry, 24 × 24 × 1 Monkhorst-Pack k-point mesh with a 4 × 4 × 1 grid in q-space are used.

To accurately calculate the Curie temperature, we have adopted the Monte Carlo simulation for the magnetization as a function of the temperature. Exchange parameters are firstly calculated, and then used to estimate the Curie temperature of the monolayer system based on Monte Carlo simulations using the VAMPIRE software package (version 5) [58,59,60,61,62]. We fit the total energies obtained from DFT calculations for four spin configurations including ferromagnetic (FM), antiferromagnetic (AFM), stripy-AFM, and zigzag-AFM via the Heisenberg spin Hamiltonian:

$$H=-J_{1}\sum_{<i,j>}\vec{S_{i}}\;\cdot \;\vec{S_{j}}-J_{2}\sum_{<<i,j>.>}\vec{S_{i}}\;\cdot \;\vec{S_{j}}-J_{3}\sum_{<<<i,j>>>}\vec{S_{i}}\;\cdot \;\vec{S_{j}}$$

Here, S is a unit vector aligned with the spin direction, and J_1_, J_2_, J_3_ are the exchange parameters for the nearest neighbors, next-nearest neighbors, and third-nearest neighbors, respectively. The Curie temperature is estimated using the Monte Carlo simulation. The simulated system for all materials consists of 11,552 spins using the EuOX crystal structure (75 × 75 supercell). This system size was verified to be sufficient for achieving negligible finite-size effects. The spins are initialized along the easiest axis [001] or [100] depending on the magnetic anisotropy energy (MAE) of the four materials. They are first thermalized via 10,000 steps to reach equilibrium, followed by 10,000 steps for averaging the thermal equilibrium magnetization at each temperature. The Curie temperature is identified by fitting the curve $M\left(T\right)={\left(1-\frac{T}{T_{c}}\right)}^{\beta }$ .

To characterize the topological properties of the band crossings in the spin-up conducting channel, we employ the following methodology. (1) Wannier Function Construction: Maximally localized Wannier functions (MLWFs) are constructed using the Wannier90 code [63]. The initial projections are chosen as Eu s, p and f orbitals, O s and p orbitals, and halogen X s and p orbitals. Convergence of the Wannierization is confirmed by verifying that the spread functional Ω is minimized and that the Wannier interpolated bands reproduce the DFT bands. (2) Weyl Point Identification and Chirality: Weyl points in the spin-up channel are first located by searching for band crossings using the WannierTools package (version 2.5.0) [63]. The topological charge (chirality) of each Weyl point is then determined by computing the Chern number on a closed one-dimensional loop enclosing the point in momentum space, defined as: $\chi =\frac{1}{2\pi }\oint_{C}A\left(k\right)\cdot dk$ , where $A\left(k\right)$ is the Berry connection, and C is the simple closed loop enclosing the 2D Weyl point.

## 3. Results and Discussion

### 3.1. Structural Property

The crystal structure belongs to the tetragonal crystal system with space group P4/nmm. Each unit cell contains two Eu atoms, two O atoms, and two halogen elements. The monolayer EuOX consists of five atomic layers, with the halogen elements located on the outermost layer. From the side view, the atomic arrangement is sequentially ordered as X-Eu-O-Eu-X. According to our calculations, the optimized lattice constants for EuOF, EuOCl, EuOBr, and EuOI are 3.620 Å, 3.854 Å, 3.928 Å, and 4.040 Å, respectively.

**Figure 1:**
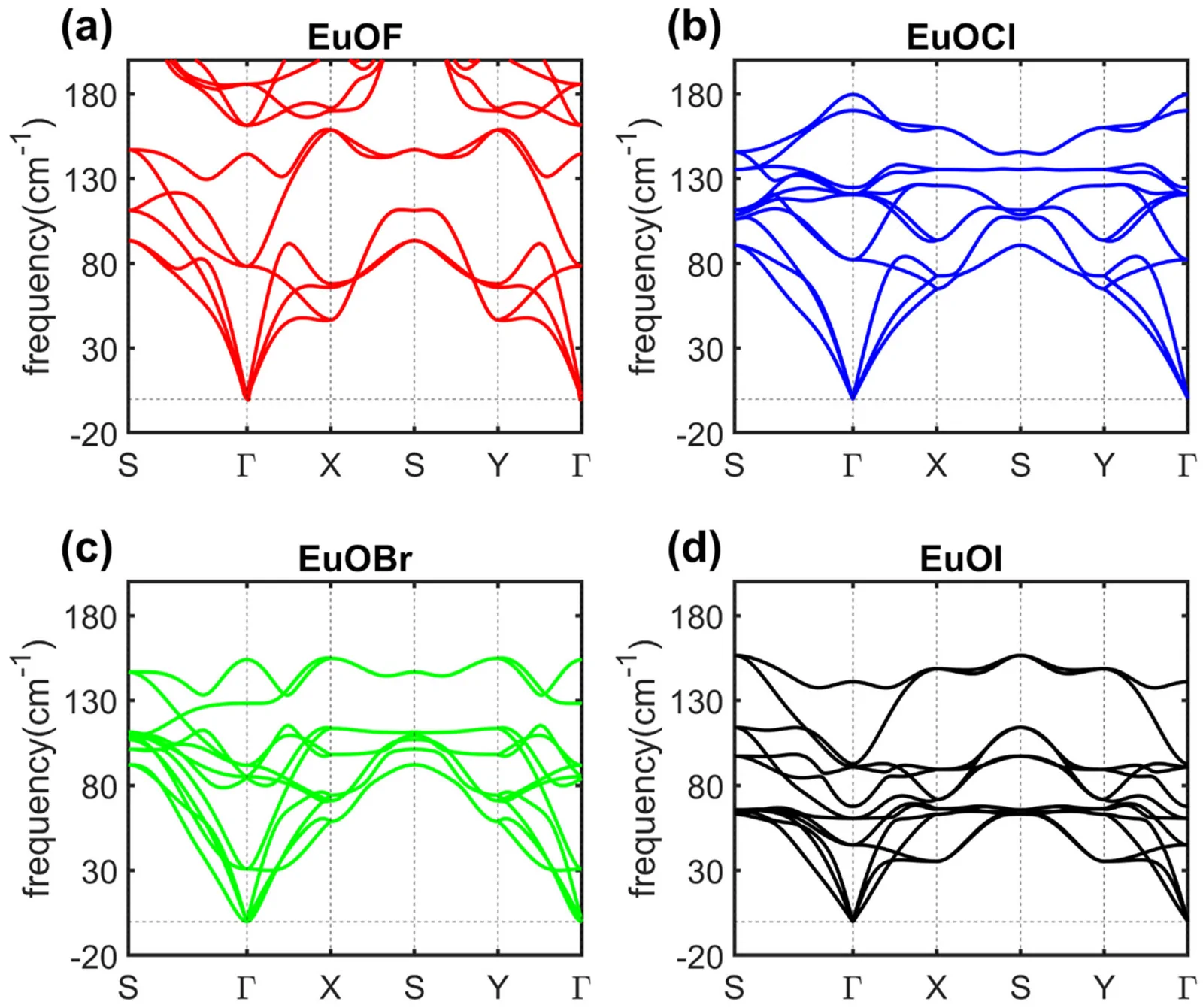
Phonon dispersion of EuOX monolayer for (**a**) EuOF, (**b**) EuOCl, (**c**) EuOBr, (**d**) EuOI.

As illustrated in Figure 1, the phonon band structures for all monolayer EuOX compounds (X = F, Cl, Br, I) exhibit no imaginary frequency across the entire Brillouin zone, which strongly indicates that these single-layer structures possess dynamical stability.

### 3.2. Cleavage Energy

**Figure 2:**
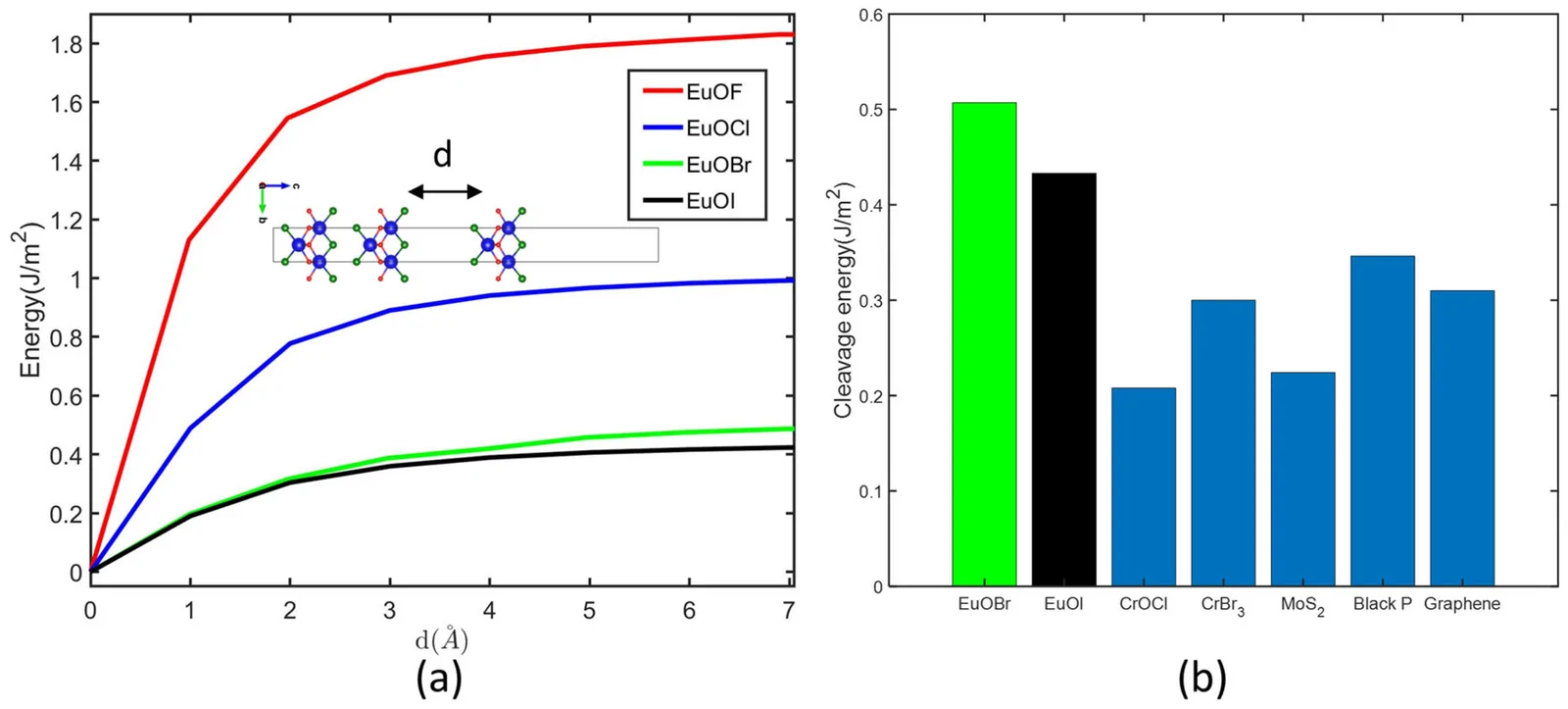
(**a**) Cleavage energies as functions of separation distance d. (**b**) Comparisons of cleavage energies between EuOX in this work and previous well-known 2D materials [58,59].

To assess the feasibility of exfoliating individual layers from the bulk EuOX materials, we calculate the corresponding cleavage energy as shown in Figure 2. A lower cleavage energy signifies easier separation. Following established protocols, we simulate the exfoliation process by introducing a layer separation distance, denoted as d, between two adjacent layers in the bulk structure (Figure 2a, inset). We employ the DFT-D2 van der Waals correction method by Grimme. The calculated cleavage energy values for EuOF, EuOCl, EuOBr, and EuOI are 1.84 J/m^2^, 0.99 J/m^2^, 0.48 J/m^2^, and 0.43 J/m^2^, respectively.

The cleavage energies for these four EuOX materials range from 0.43 J/m^2^ to 1.84 J/m^2^. Notably, the cleavage energies of EuOBr, EuOI, and other analogous systems such as CrOCl (0.21 J/m^2^) [64], CrBr3 (0.30 J/m^2^) [64], Black Phosphorus (0.35 J/m^2^), MoS_2_ (0.22 J/m^2^), and graphene (0.31 J/m^2^) [64] fall within relatively low values. This suggests that experimentally isolating single layers from bulk EuOX materials is likely achievable, as their cleavage energies are comparable to those of other well-established exfoliable materials like MoS_2_ and graphene.

### 3.3. Magnetic Property

**Figure 3:**
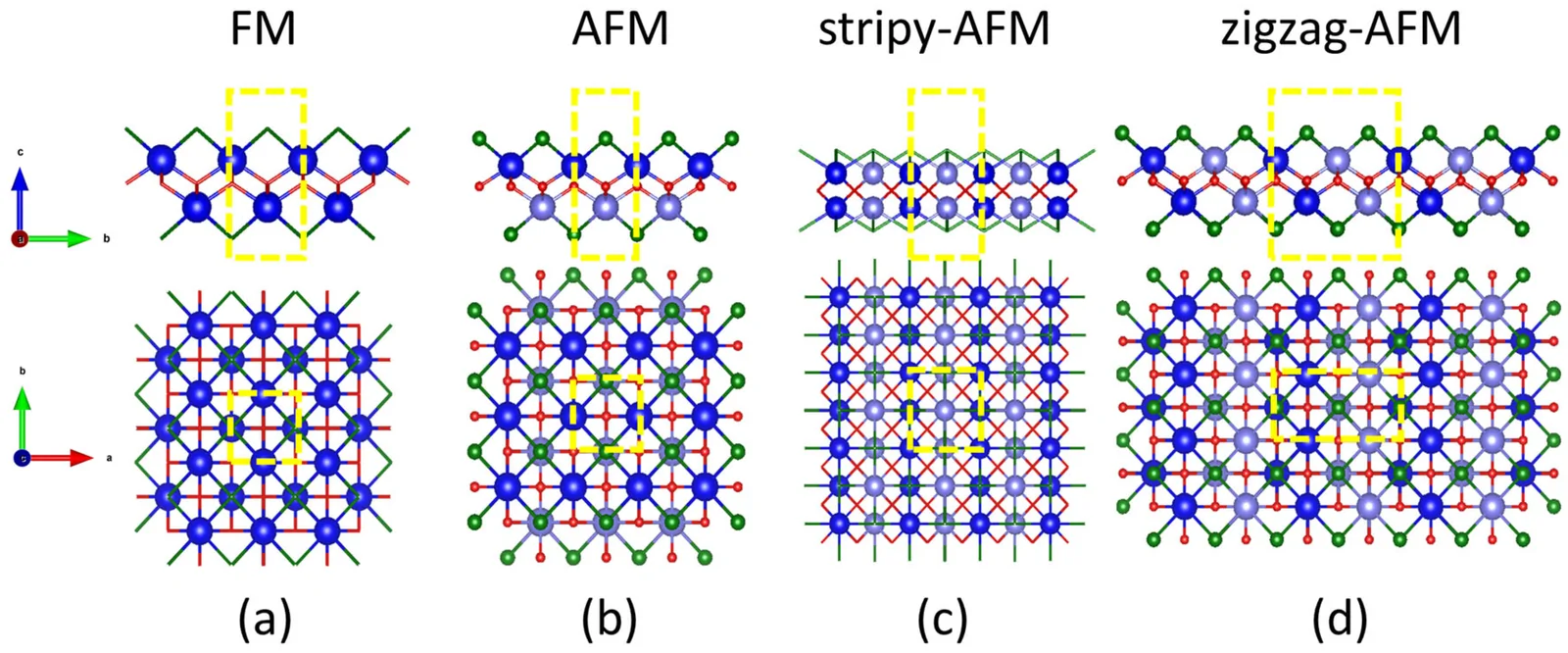
Magnetic configurations of the EuOX monolayer considered in this work: (**a**) ferromagnetic (FM), (**b**) antiferromagnetic (AFM), (**c**) stripy-AFM, and (**d**) zigzag-AFM states. The darker and brighter blue spheres represent spin-up and spin-down Eu atoms, respectively. The yellow rectangle denotes the unit cell.

To determine the magnetic exchange interactions, we considered four distinct magnetic configurations, including ferromagnetic (FM), antiferromagnetic (AFM), stripy-AFM, and zigzag-AFM, as illustrated in Figure 3. These four configurations are chosen because they constitute a complete and linearly independent set of spin orderings on the square Eu sublattice, providing the minimum number of equations required to uniquely determine the three independent exchange parameters J_1_, J_2_, and J_3_. The exchange parameters (J_1_, J_2_, J_3_) were calculated using the energy difference method. The magnetic energy expressions for EuOX monolayers are given by:

$$\begin{array}{c} E_{fm}=E_{0}-4J_{1}-4J_{2}-4J_{3}\\ E_{afm}=E_{0}+4J_{1}-4J_{2}-4J_{3}\\ E_{stripy\;afm}=E_{0}+4J_{2}-4J_{3}\\ E_{zigzag\;afm}=E_{0}+4J_{3} \end{array}$$

Our calculations quantified the magnetic exchange interactions within the EuOX monolayers (X = F, Cl, Br, I) using first-principles methods. The results for the Heisenberg model parameters (J_1_, J_2_, J_3_) and the estimated Curie temperatures (T_C_) are summarized in Table 1. The nearest-neighbor exchange coupling J_1_ exhibits positive values of 24, 29, 30, and 24 meV for EuOF, EuOCl, EuOBr, and EuOI, respectively, confirming strong ferromagnetic interactions. The next-nearest-neighbor coupling J_2_ also shows positive values of 16, 20, 21, and 16 meV, respectively, further supporting the ferromagnetic nature. Although the J_3_ value of −6 meV is negative for EuOF, EuOCl, and EuOBr, and is 0.8 meV for EuOI, their magnitudes are significantly smaller than J_1_ and J_2_.

**Figure 4:**
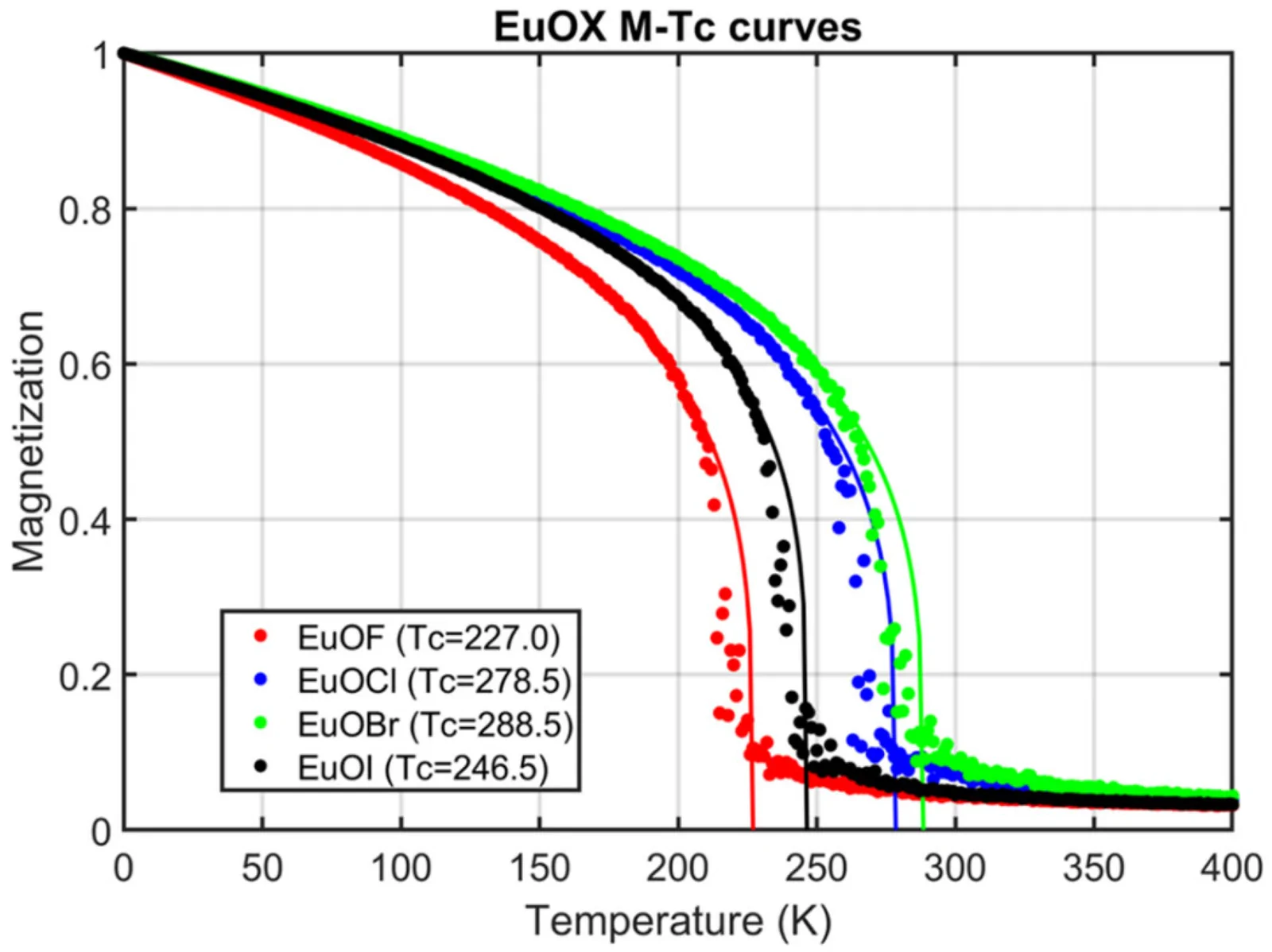
Monte Carlo simulations on Curie temperatures of EuOX. The solid lines are fitting curves.

As mentioned previously, both the exchange parameters J_1_ and J_2_ show strong ferromagnetic couplings with an order of magnitude much larger than the negligible antiferromagnetic coupling J_3_, thus it is a good approximation to estimate the Curie temperature based on the dominant nearest-neighbor exchange parameter J_1_. As show in Figure 4, the calculated Curie temperatures are 227 K for EuOF, 278 K for EuOCl, 289 K for EuOBr, and 247 K for EuOI. Significantly, EuOBr exhibits the highest Curie temperature (289 K), bringing it closest to room temperature among the four compounds. This high Curie temperature, combined with the previously determined stability and exfoliability, positions EuOBr as a particularly promising candidate for room-temperature spintronic applications.

### 3.4. Electronic Structure

**Figure 5:**
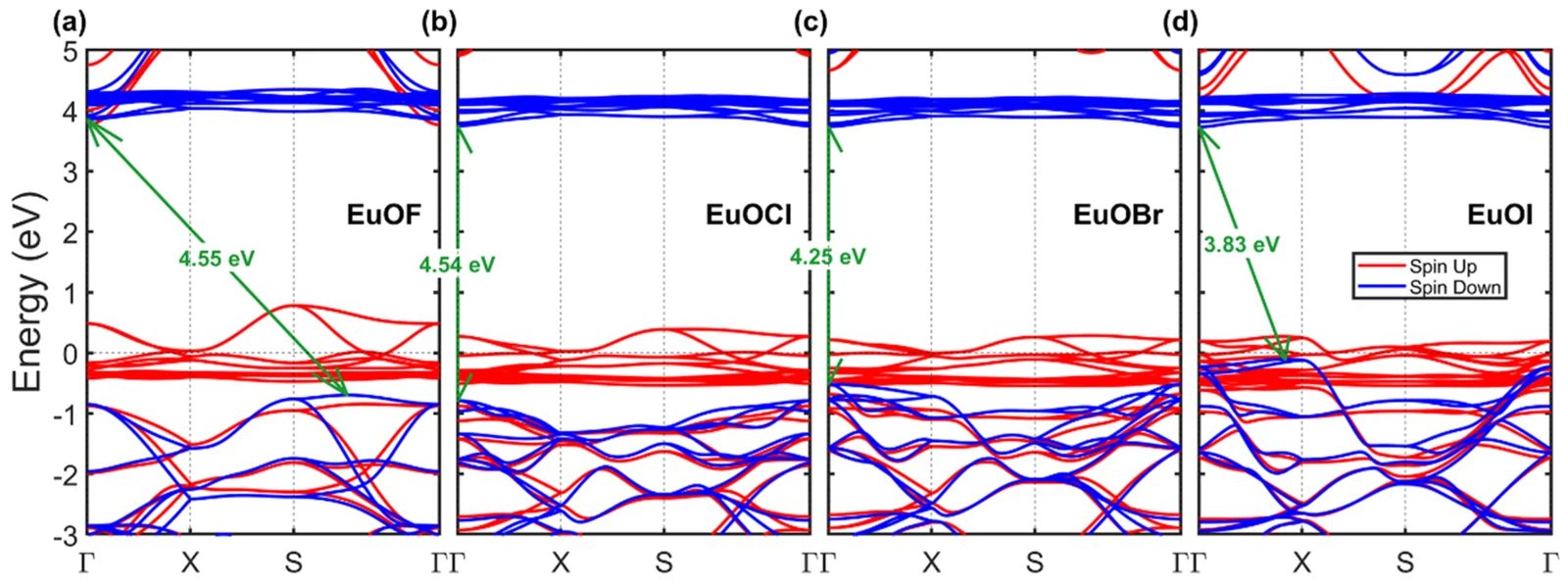
Electronic band structure of (**a**) EuOF, (**b**) EuOCl, (**c**) EuOBr, (**d**) EuOI. The spin-up and spin-down bands are colored by red and blue, respectively. The green arrows indicate the energy band gaps in the spin-down channel.

The electronic band structures of EuOX monolayers (X = F, Cl, Br, I) have been systematically investigated. Our findings reveal that the spin-up channel exhibits a metallic band structure, while the spin-down channel demonstrates an insulating band structure as shown in Figure 5. This observation unequivocally confirms that the EuOX series functions as two-dimensional (2D) half-metals, where all conducting electrons maintain identical spin orientations with 100% spin polarization at the Fermi level.

The insulating gaps in the spin-down channel of these compounds as shown in Figure 5 are 4.55 eV for EuOF, 4.54 eV for EuOCl, 4.25 eV for EuOBr, and 3.83 eV for EuOI. Significantly, EuOCl and EuOBr demonstrate a direct band gap, while the other compounds exhibit indirect band gaps (Figure 5). To further elucidate the origin of the direct/indirect band gap character, we perform a series of constrained lattice calculations in which the lattice structure of a given material is substituted by that of a neighboring compound in the series. Specifically, the lattice constant 3.620 Å of EuOF is replaced by 3.854 Å of EuOCl. Then the expanded lattice parameters change the band gap character of EuOF from an indirect to a direct gap. Similarly, EuOI computed with the EuOBr lattice constant (3.928 Å, compressed from its original 4.040 Å) also recovers a direct band gap. These results demonstrate that the direct/indirect band gap transition is primarily related to the lattice structural.

**Figure 6:**
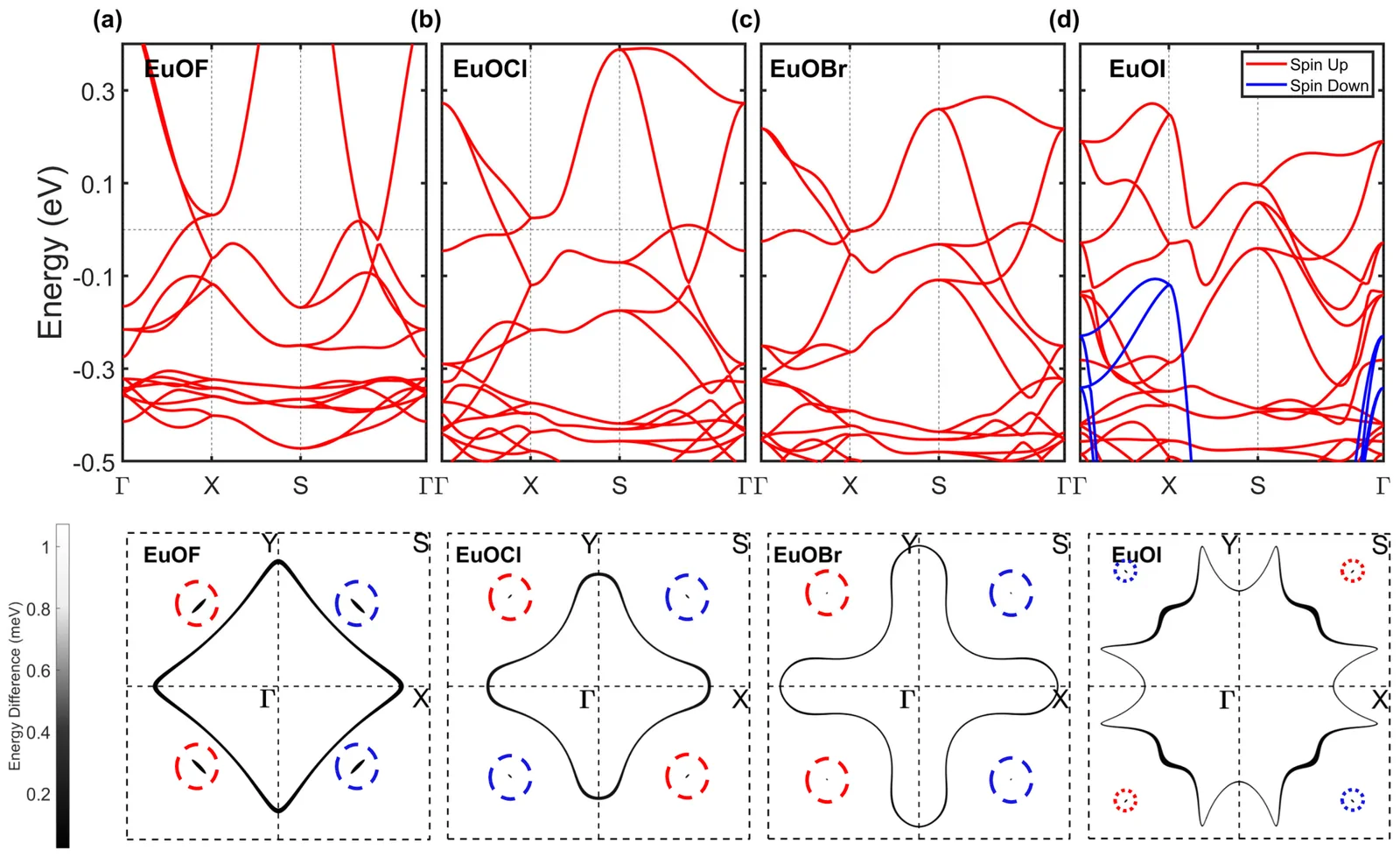
The nodal line band structure, contour, and Weyl points in EuOX compounds without spin–orbit coupling: (**a**) EuOF (**b**) EuOCl (**c**) EuOBr (**d**) EuOI. The blue (red) circles denote Weyl points with chirality χ=−1 (+1) .

Notably, band crossings near the Fermi level can be seen in the metallic spin-up channel. To clarify the band topology, Figure 6 illustrates the band structure details around the Fermi level. These crossings indicate that EuOF, EuOCl, EuOBr, and EuOI possess nontrivial topological band structures, a phenomenon that will be discussed in detail below. Together, these results highlight the distinct electronic properties of EuOX monolayers, making them promising candidates for spintronic applications. Four Weyl points are identified in the spin-up conducting channel, carrying chiralities as indicated in Figure 6.

**Figure 7:**
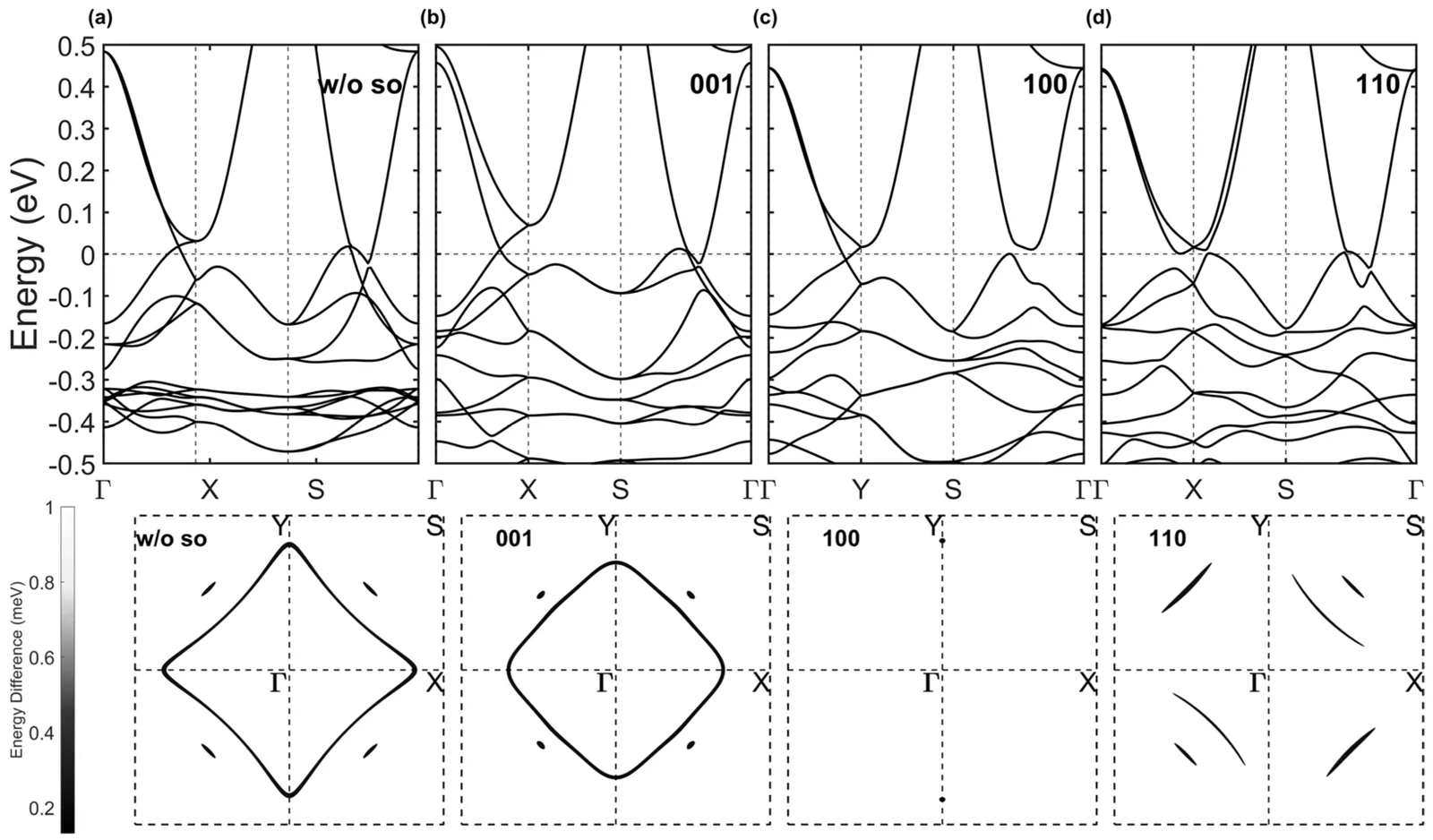
Impact of spin–orbit coupling (SOC) on the band structure (**upper panel**) and nodal line/Weyl point distributions (**lower panel**) of EuOF under different spin orientations. The four panels represent: (**a**) without spin–orbit coupling (w/o SOC), (**b**) with spin-orbit coupling and spin direction along [001], (**c**) with spin direction along [100], (**d**) with spin direction along [110].

**Figure 8:**
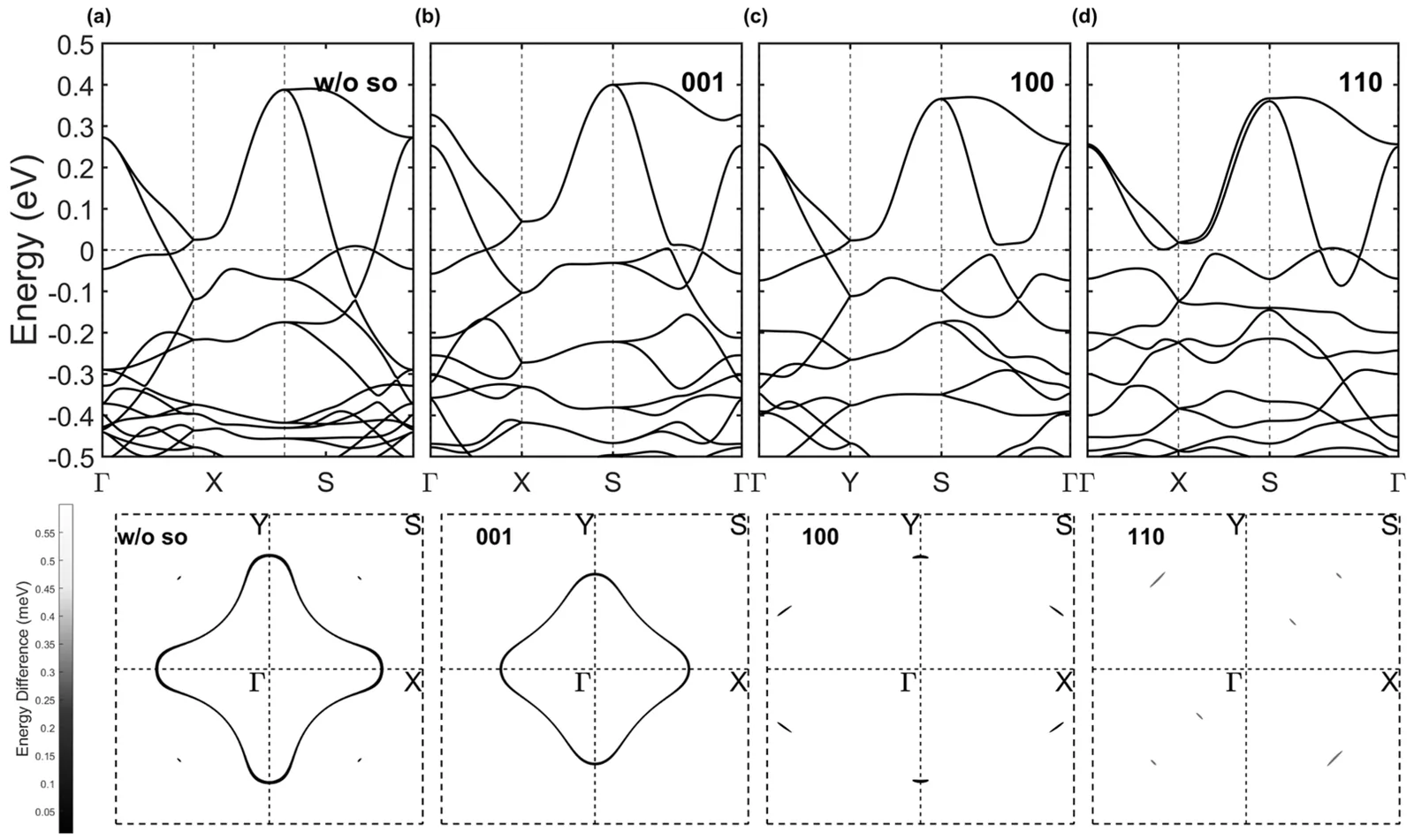
Impact of spin–orbit coupling (SOC) on the band structure (**upper panel**) and nodal line/Weyl point distributions (**lower panel**) of EuOCl under different spin orientations. The four panels represent: (**a**) without spin–orbit coupling (w/o SOC), (**b**) with spin-orbit coupling and spin direction along [001], (**c**) with spin direction along [100], (**d**) with spin direction along [110].

**Figure 9:**
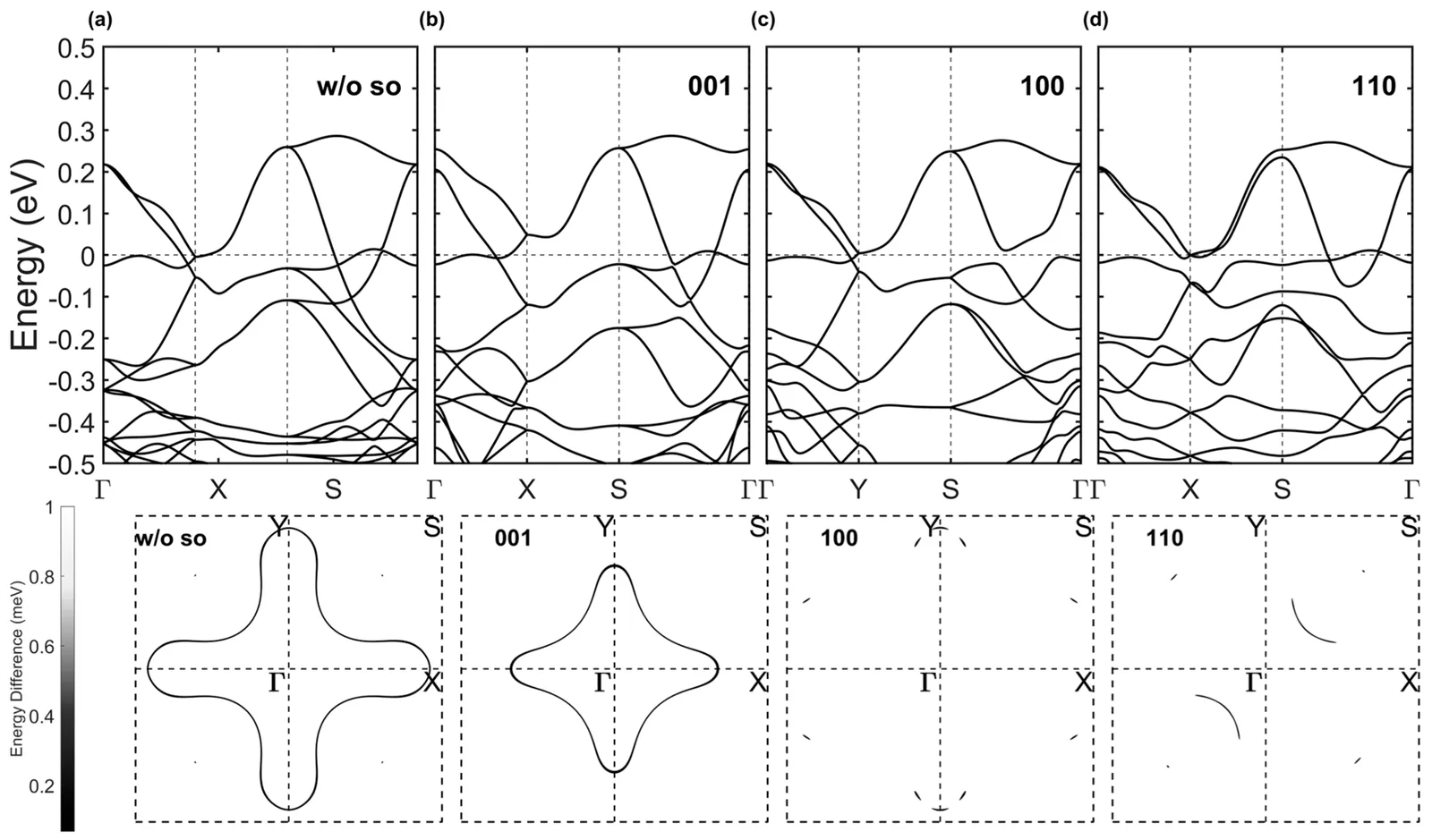
Impact of spin–orbit coupling (SOC) on the band structure (**upper panel**) and nodal line/Weyl point distributions (**lower panel**) of EuOBr under different spin orientations. The four panels represent: (**a**) without spin–orbit coupling (w/o SOC), (**b**) with spin–orbit coupling and spin direction along [001], (**c**) with spin direction along [100], (**d**) with spin direction along [110].

**Figure 10:**
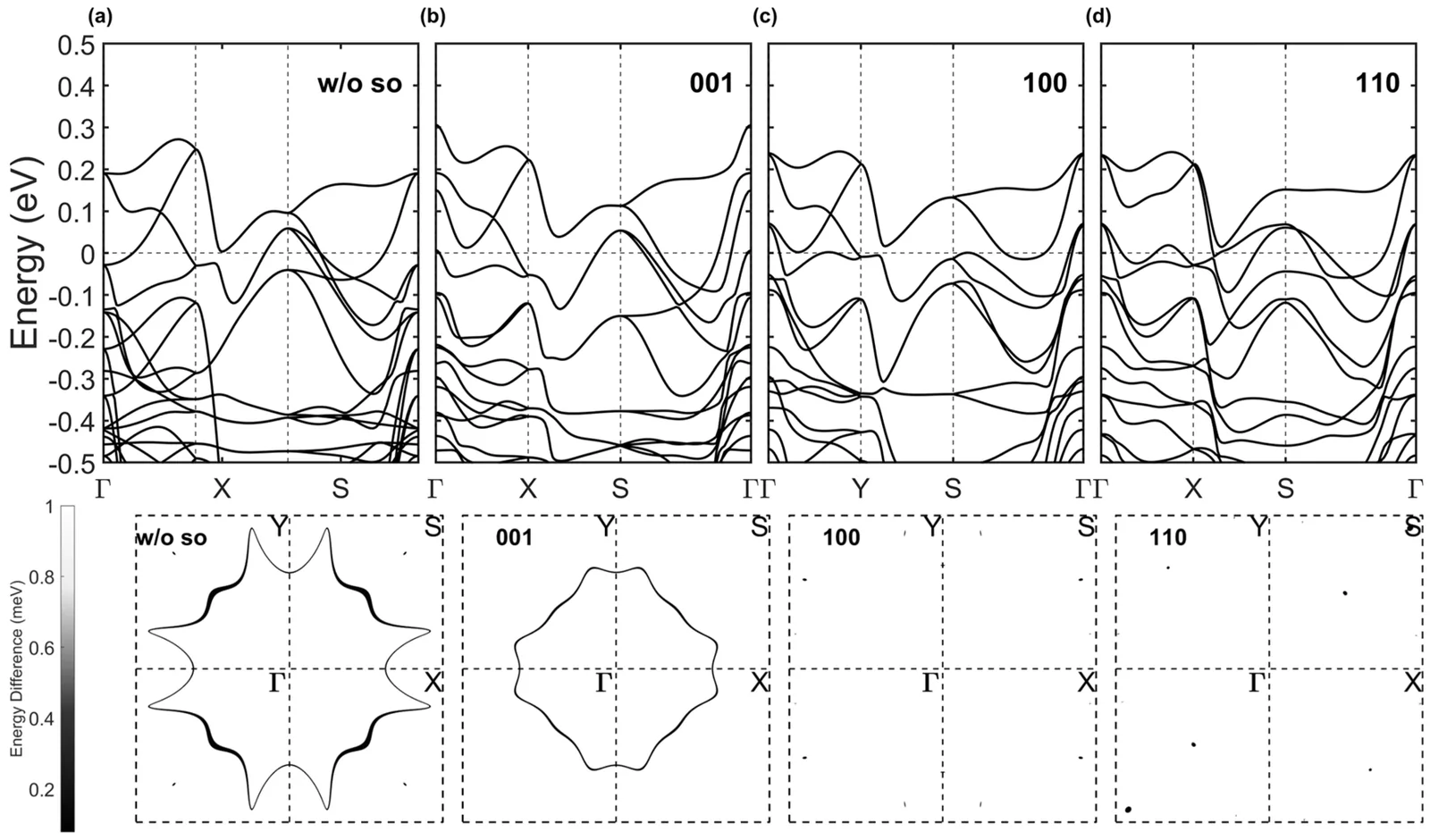
Impact of spin–orbit coupling (SOC) on the band structure (**upper panel**) and nodal line/Weyl point distributions (**lower panel**) of EuOI under different spin orientations. The four panels represent: (**a**) without spin–orbit coupling (w/o SOC), (**b**) with spin–orbit coupling and spin direction along [001], (**c**) with spin direction along [100], (**d**) with spin direction along [110].

To further explore the impact of spin–orbit coupling and spin orientation on the electronic structure, we systematically compare the band structures under various conditions as shown in Figure 7, Figure 8, Figure 9 and Figure 10 for EuOF, EuOCl, EuOBr, EuOI, respectively. Our analysis reveals that the nodal lines in these materials appear smoother under spin–orbit coupling. Additionally, owing to the stronger spin–orbit coupling in heavier elements, the atomic number of X in the EuOX compounds (EuOF, EuOCl, EuOBr, EuOI) directly correlates with the change induced by spin–orbit coupling on the nodal lines.

As depicted in Figure 7, Figure 8, Figure 9 and Figure 10, we identified significant variations in the band structures near the Fermi level under different spin orientations. These observations imply the high magnetic anisotropy energy of these materials. Specifically, when the spin moment is oriented along the [100] direction, the original nodal lines, which lack Mz symmetry, completely disappear. In this configuration, four of the compounds exhibit Weyl points near the Y point protected by the Mx symmetry. When the spin moment is oriented along the [110] direction, Weyl points appear in the band structure.

### 3.5. Strong Correlation DFT+U

**Figure 11:**
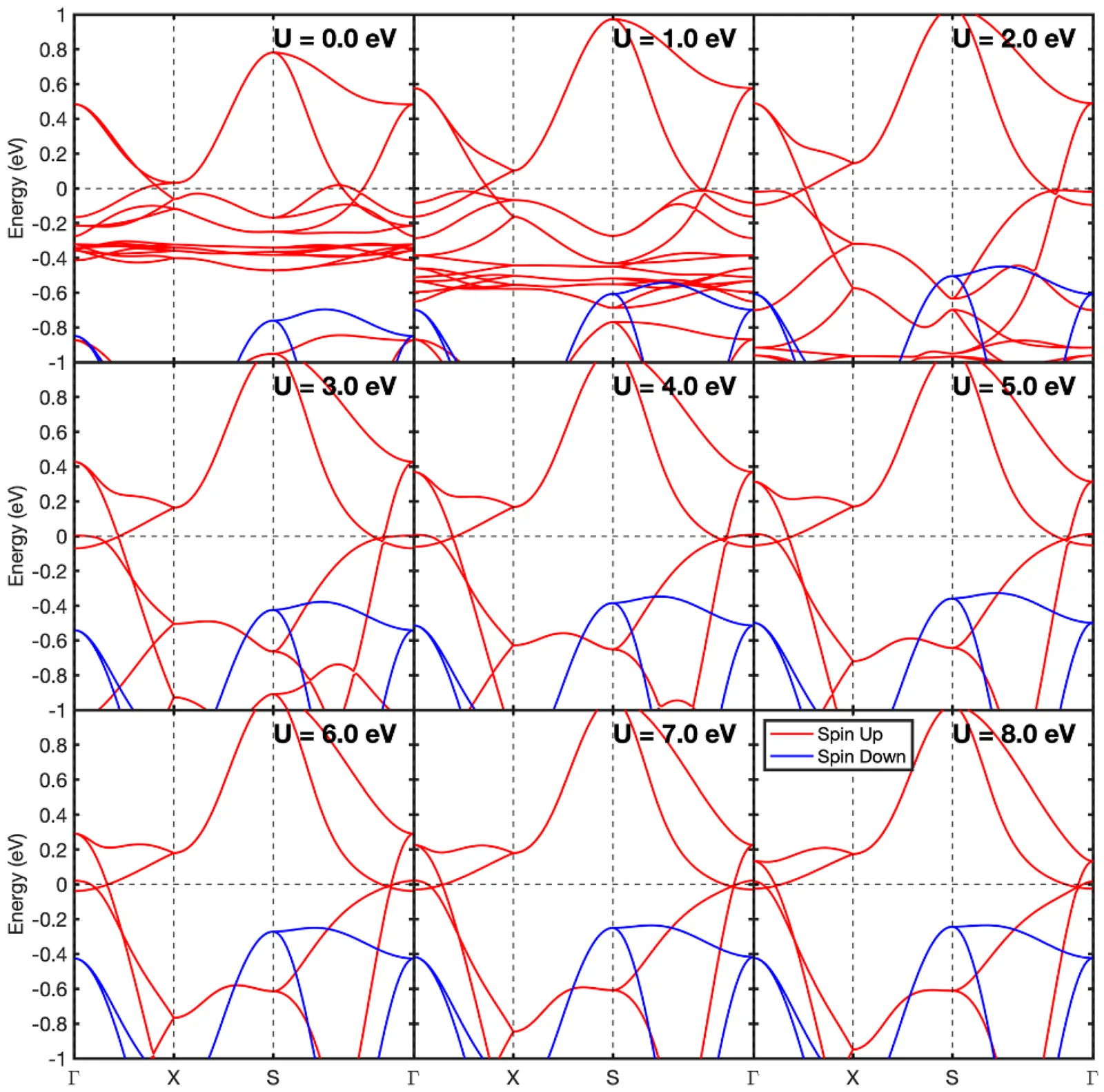
Evolution of the band structure in EuOF as a function of the on-site Coulomb repulsion parameter U, ranging from 0 to 8 eV. The diagrams highlight the robust half-metallic band structure despite the significant changes in band dispersion affected by on-site U.

**Figure 12:**
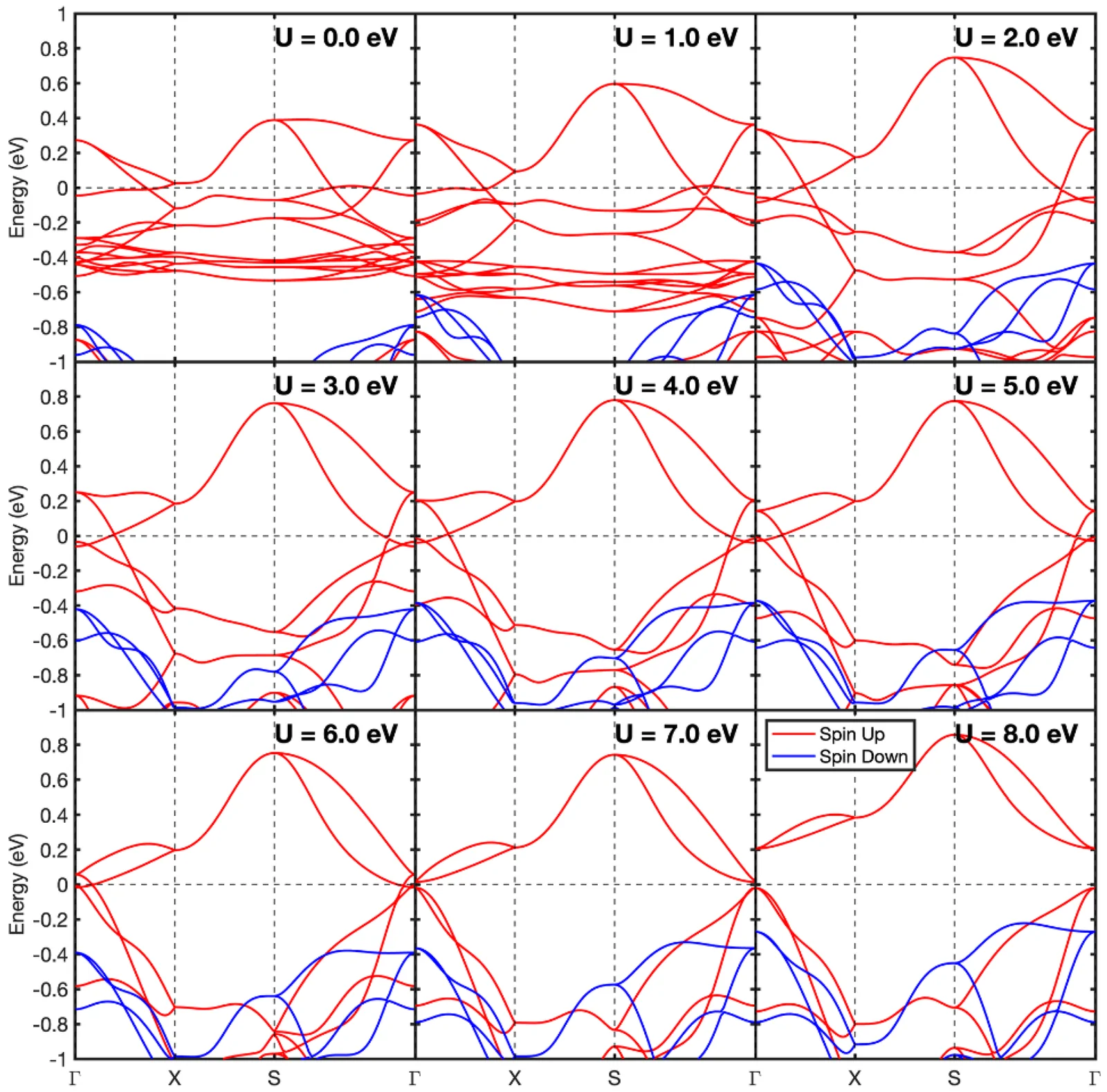
Evolution of the band structure in EuOCl as a function of the on-site Coulomb repulsion parameter U, ranging from 0 to 8 eV. The diagrams highlight a metal insulator phase transition from half-metal to semi-half-metal at U = 7 eV, and then to magnetic semiconductor with U = 8 eV, accompanied by significant changes in band dispersion and gap opening.

**Figure 13:**
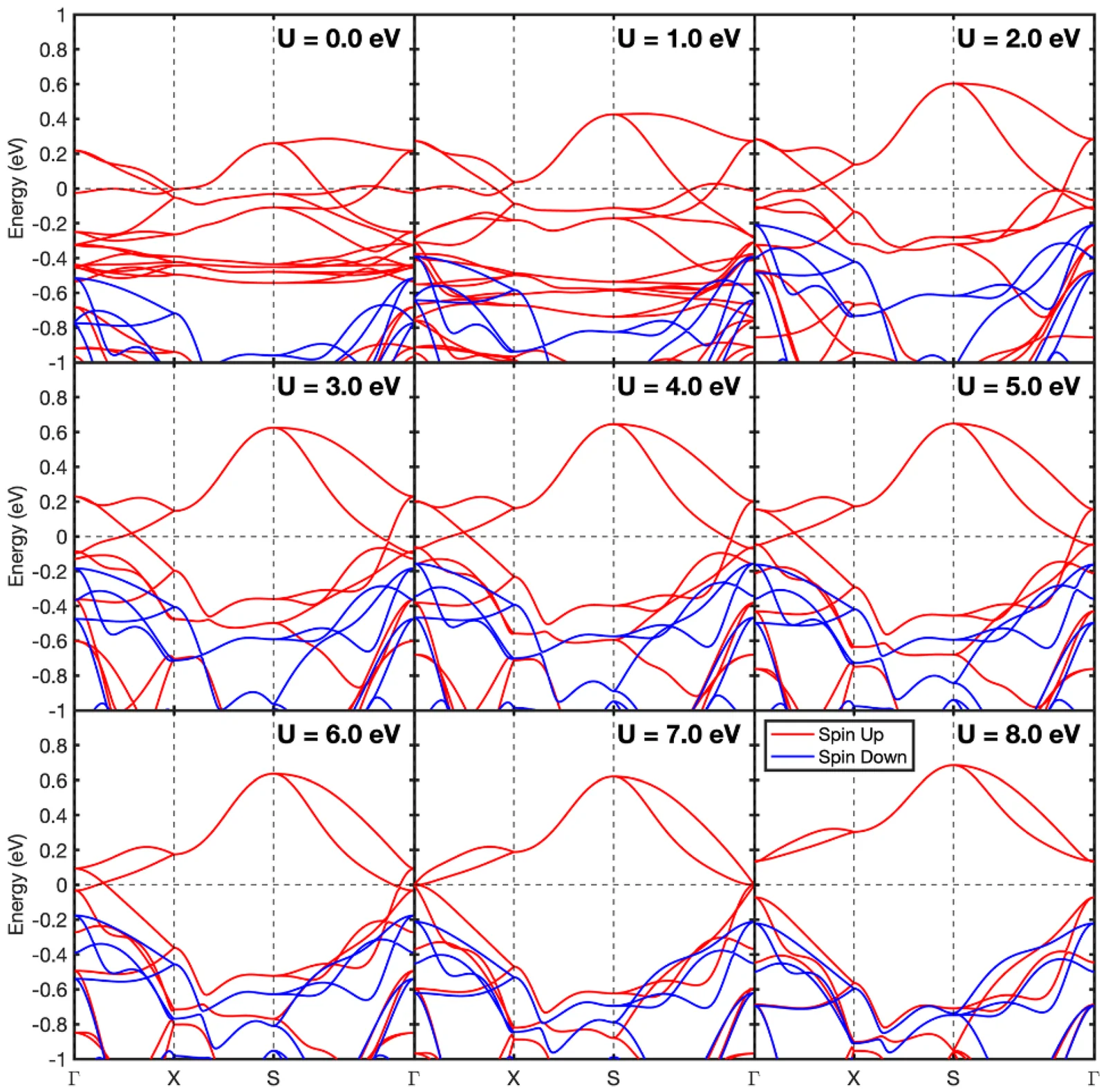
Evolution of the band structure in EuOBr as a function of the on-site Coulomb repulsion parameter U, ranging from 0 to 8 eV. The diagrams highlight a metal insulator phase transition from half-metal to semi-half-metal, and then to magnetic semiconductor around U = 7 eV, accompanied by significant changes in band dispersion and gap opening.

**Figure 14:**
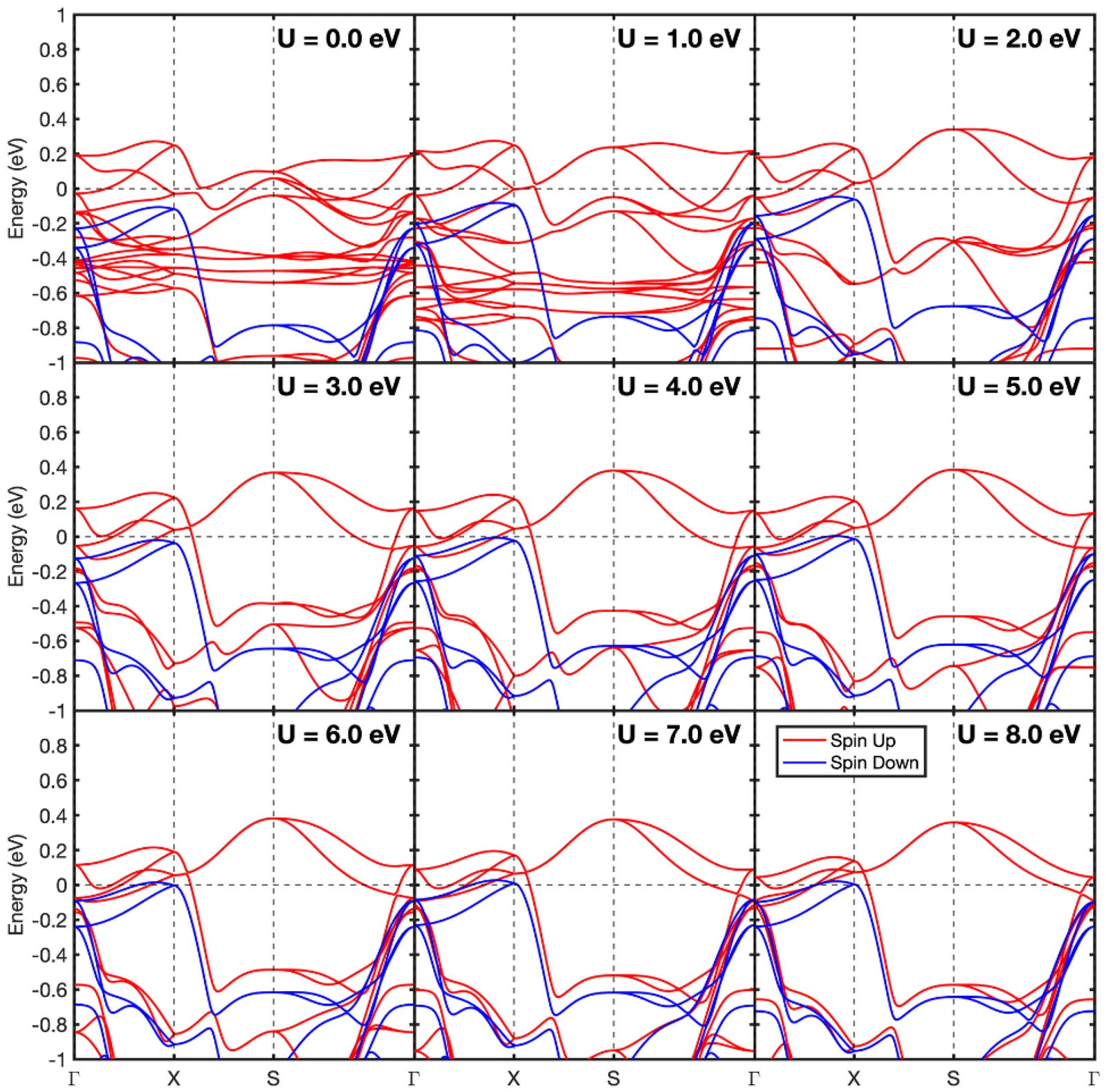
Evolution of the band structure in EuOI as a function of the on-site Coulomb repulsion parameter U, ranging from 0 to 8 eV. The diagrams highlight a half-metal to magnetic metal phase transition with U beyond 4 eV, ruining the half-metallic 100% spin polarization at the Fermi level, though the spin polarization at the Fermi level remains high.

To pursue the strong correlation effect, we have performed DFT+U [51] calculations for EuOX to systematically examine the influence of the on-site Coulomb repulsion Hubbard U parameter on the electronic structures of the EuOX monolayers (X = F, Cl, Br, I). The results, visualized in Figure 11, Figure 12, Figure 13 and Figure 14, reveal distinct material-dependent responses as discussed below.

For EuOF, Figure 11 demonstrates that varying Hubbard U (0 to 8 eV) induces shifts in band crossings near the Fermi level. Notably, EuOF maintains its half-metallic character across this entire range of U values, confirming its robustness in half-metallicity to electron correlation effects within the studied parameter space.

EuOCl, as shown in Figure 12, undergoes a phase transition at U = 7.0 eV. At this critical U value, the material transitions from a half-metallic to a semi-half-metallic state, indicating a significant change in its topological properties where nodal lines likely emerge or modify. Further increasing U to 8.0 eV causes EuOCl to lose its metallic character entirely, leading to a metal insulator transition from a half-metal to a magnetic semiconductor.

EuOBr exhibits a similar transition at U = 7.0 eV, transforming from a half-metallic to a semi-half-metallic phase as shown in Figure 13. This transition is characterized by the disappearance of nodal lines and the formation of a four-fold degenerate crossing at the Γ point. Increasing U further to 8.0 eV removes EuOBr’s metallic character and transforms it to a magnetic semiconducting phase.

In the case of EuOI, Figure 14 reveals a different behavior. As Hubbard U increases from 0 to 8 eV, flat bands experience vertical shifts, and the original nodal lines and Weyl points undergo energetic and positional changes. Crucially, EuOI maintains its half-metallic nature with U values ranging from 0 to 4 eV. As U is beyond 4 eV, the highest occupied spin-down band reaches the Fermi level, resulting in magnetic metal behavior with the half-metallic phase destroyed and no more 100% spin polarization at the Fermi level. However, the spin polarization at the Fermi level remains high for these magnetic metals as can be seen in the figures.

**Figure 15:**
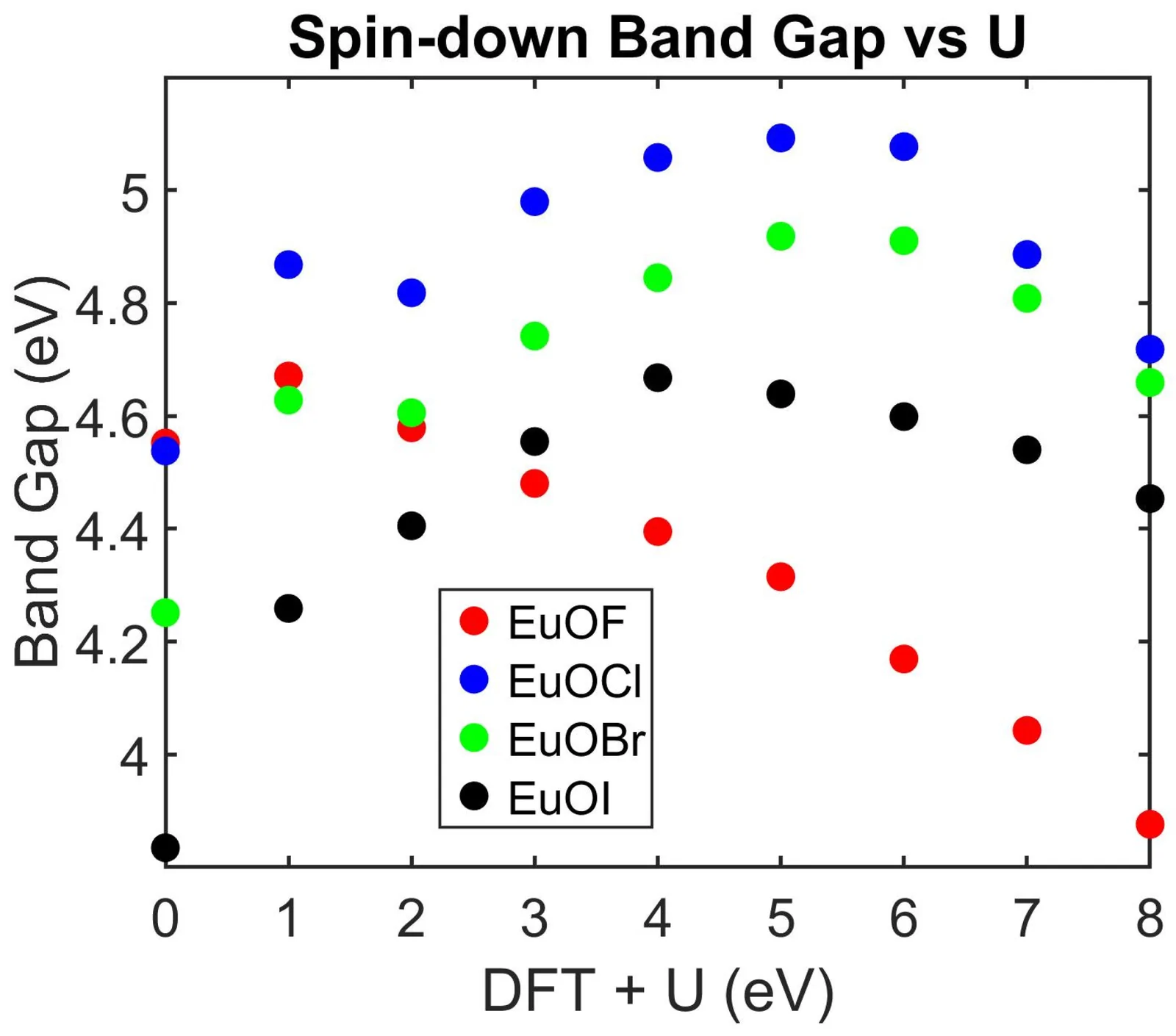
The Hubbard U dependence of the energy band gap in the spin-down channel of EuOX monolayer.

**Figure 16:**
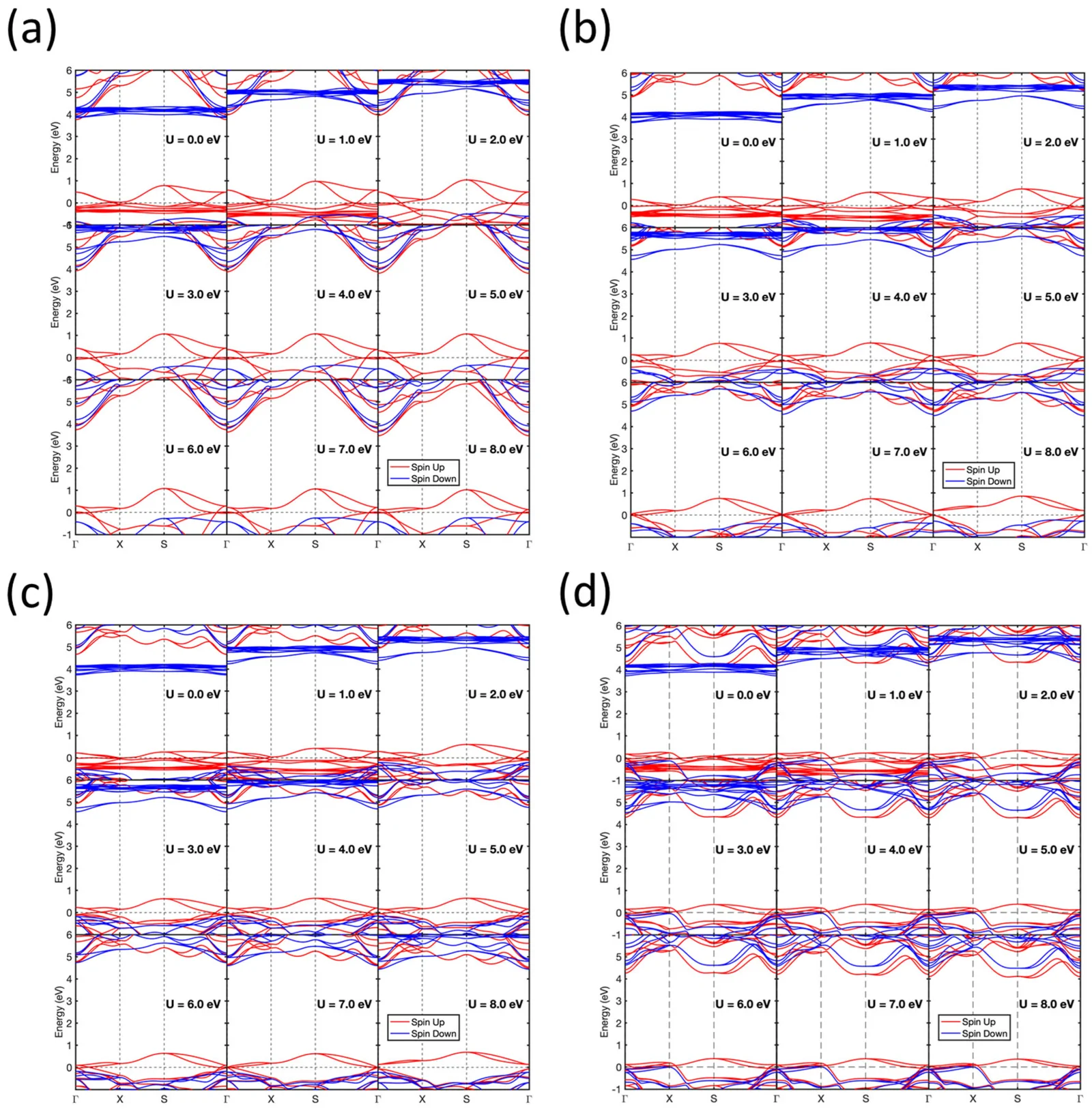
The evolution of the band structure of (**a**) EuOF, (**b**) EuOCl, (**c**) EuOBr, and (**d**) EuOI as a function of the on-site Coulomb repulsion parameter U. To emphasize the influence of the spin-down channel on varying U values, a broad energy range is displayed for each compound.

Complementing these findings, Figure 15 analyzes the U-dependence of the spin-down band gaps. EuOCl, EuOBr, and EuOI display analogous trends: the band gaps initially increase and then exhibit a gradual decrease. This behavior is attributed to the combined effect of Hubbard U shifting flat bands away from the Fermi level (increasing the gap) and potentially altering band overlaps at higher U values (causing the subsequent decrease). EuOF demonstrates a similar trend, albeit with a smaller magnitude as shown in Figure 16.

**Figure 17:**
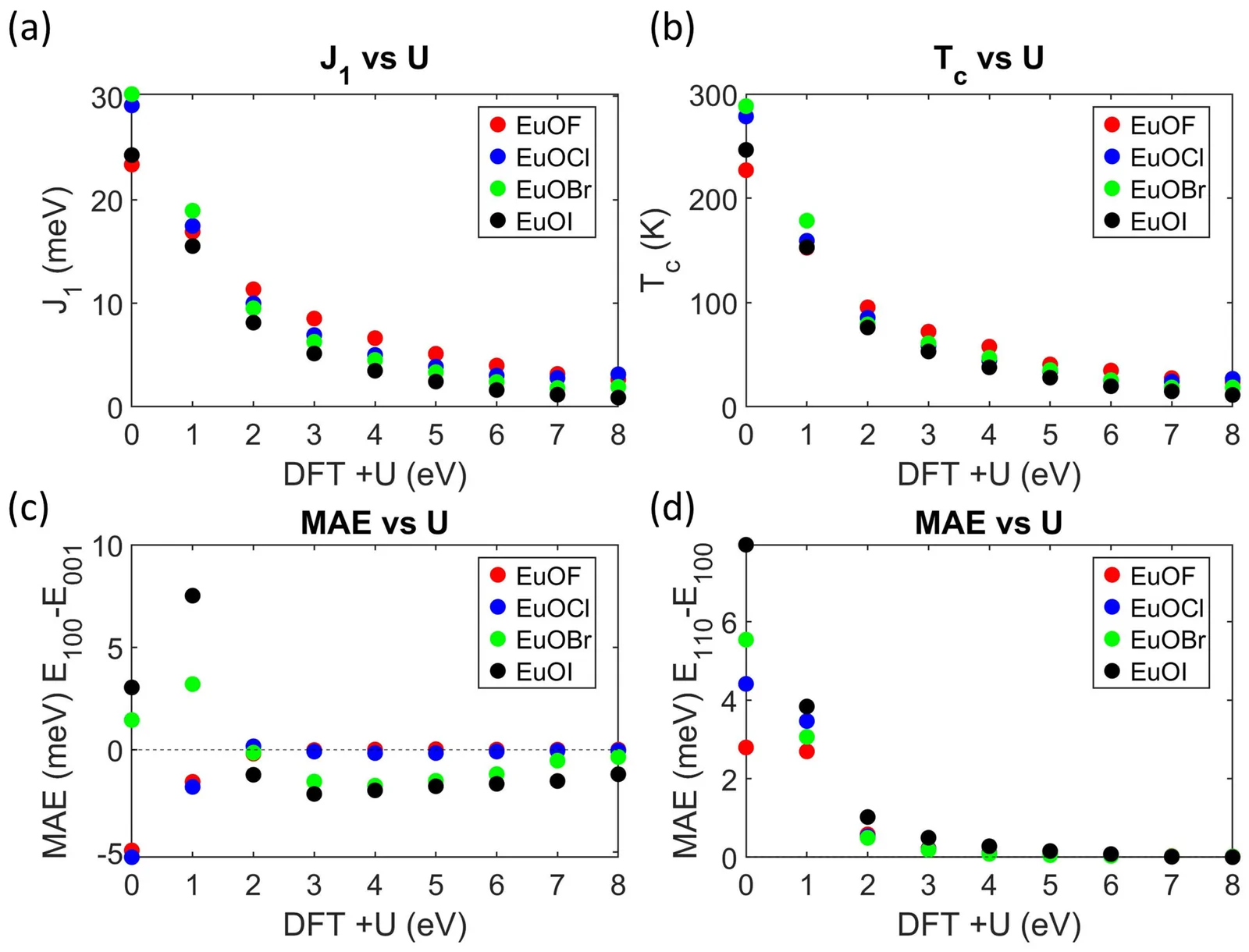
The Hubbard U dependence of (**a**) exchange parameter J_1_, (**b**) Curie temperature, (**c**,**d**) magnetic anisotropy energy (MAE) difference between different spin directions of EuOX monolayers.

In Figure 17, we summarize the U-dependence of the nearest-neighbor exchange parameter J_1_, Curie temperature T_C_, magnetic anisotropy energy (MAE) E_100_−E_001_, and MAE E_110_−E_100_. To facilitate computational analysis, we focused on the nearest-neighbor exchange energy J_1_ and employed Monte Carlo simulations to estimate the Curie temperature (T_C_). The results demonstrated that J_1_ decreases exponentially with increasing Hubbard U across all four materials. Correspondingly, the Curie temperature decreases to approximately 10 K for all compounds. Notably, even within the typical U range for europium (4–8 eV) [43,53,54,55,56], all four EuOX materials retain their ferromagnetic characteristics.

Regarding magnetic anisotropy energy (MAE), at U = 0.0 eV, EuOBr and EuOI exhibit an out-of-plane magnetic moment direction (PMA), while EuOF and EuOCl show an in-plane (100) orientation. When U increases to 2.0 eV, the easy axis for EuOBr and EuOI shifts to the (100) direction. In contrast, EuOF and EuOCl maintain negligible magnetic anisotropy, with MAE approaching 0 eV.

Our investigation systematically examines the influence of the Hubbard U parameter on the electronic structures of the EuOX monolayers (X = F, Cl, Br, I). The results visualized in Figure 11, Figure 12, Figure 13, Figure 14, Figure 15, Figure 16 and Figure 17 reveal distinct material-dependent responses and highlight the complex interplay between electron correlation (captured by Hubbard U) and the intrinsic electronic properties of EuOX materials, providing crucial insights for predicting and designing functional two-dimensional magnetic materials.

To facilitate experimental validation of our theoretical results, we propose the following experiments to examine our predictions. Scanning tunneling microscopy (STM) can be utilized for precise structural measurements to confirm the atomic-scale morphology and lattice parameters of the exfoliated EuOX monolayers. Furthermore, angle-resolved photoemission spectroscopy (ARPES) is ideally suited to map the electronic band structure, providing direct observation of the band crossings near the Fermi level. This technique would allow for the unambiguous verification of the predicted Weyl points and nodal lines. Finally, magneto-optic Kerr effect (MOKE) microscopy offers a highly sensitive, non-destructive approach to confirm the intrinsic 2D ferromagnetism. By quantifying the magnetic remanence and surface magnetic anisotropy as a function of temperature, MOKE can effectively validate the predicted high T_c_.

## 4. Conclusions

In summary, we propose the EuOX (X = F, Cl, Br, I) family as a novel series of two-dimensional magnetic topological materials, whose properties have been systematically studied with the Hubbard U parameter taken into consideration. Our calculations demonstrate that varying U significantly alters the band dispersion and topological features of these materials, including the presence and characteristics of Weyl points and nodal lines. Furthermore, while EuOF remains a half-metal, we observe distinct metal-to-insulator phase transitions, specifically, from half-metal, semi-half-metal, to magnetic semiconductor in EuOCl and EuOBr, and from half-metal to magnetic metal in EuOI, as a function of changing Hubbard U values. These transitions highlight the sensitivity of the electronic and magnetic properties to electron correlation effects. Crucially, the electronic band structure also exhibits dependence on the direction of magnetization. Different spin orientations lead to distinct band configurations, further enriching the material behaviors. Collectively, these findings establish the EuOX materials as promising platforms for exploring and developing applications in the field of topological spintronics, offering tunable magnetic, semiconducting, and topological properties suitable for future technological advancements.

## Figures and Tables

**Table 1:** Exchange parameters J_1_, J_2_, J_3_ and the estimated Curie temperatures T_C_ for EuOX.

Compound	J_1_ (meV)	J_2_ (meV)	J_3_ (meV)	T_C_ (K)
EuOF	24	16	−6	227.0
EuOCl	29	20	−6	278.5
EuOBr	30	21	−6	288.5
EuOI	24	16	0.8	246.5

## Data Availability

The original contributions presented in this study are included in the article. Further inquiries can be directed to the corresponding author.
