# Correction: Hung, S.-H.; Jeng, H.-T. Topological Weyl and Nodal Line Half-Metals in Two-Dimensional van der Waals Material EuOX (X = F, Cl, Br, I). *Materials* 2026, *19*, 2154

**DOI:** 10.3390/ma19183829

**Published:** 2026-09-09

**Authors:** Sheng-Hsiung Hung, Horng-Tay Jeng

**Affiliations:** 1 Department of Physics, National Tsing Hua University, Hsinchu 30013, Taiwan; 2 Physics Division, National Center for Theoretical Sciences, Taipei 10617, Taiwan; 3 Institute of Physics, Academia Sinica, Taipei 11529, Taiwan; 4 Research Center for Semiconductor Materials and Advanced Optics, Chung Yuan Christian University, Taoyuan 32031, Taiwan

In the original publication [1], some content in Figure 9 overlapped with that in Figure 8. The corrected Figure 9 appears below. The authors state that the scientific conclusions are unaffected. This correction was approved by the Academic Editor. The original publication has also been updated.

## Figures and Tables

**Figure 9:**
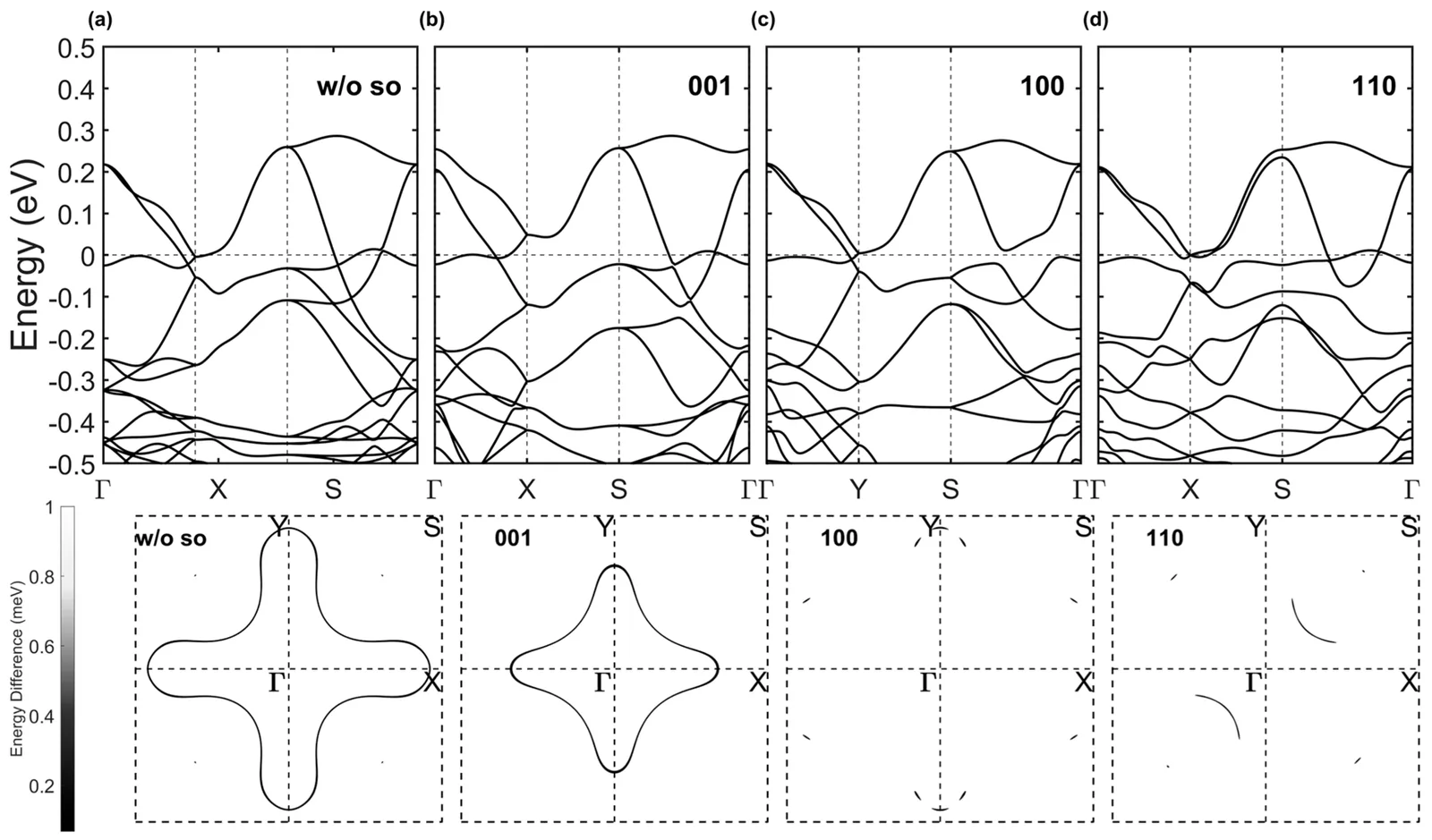
Impact of spin–orbit coupling (SOC) on the band structure (**upper panel**) and nodal line/Weyl point distributions (**lower panel**) of EuOBr under different spin orientations. The four panels represent: (**a**) without spin–orbit coupling (w/o SOC), (**b**) with spin–orbit coupling and spin direction along [001], (**c**) with spin direction along [100], (**d**) with spin direction along [110].
